# Genome-wide analysis of long noncoding RNAs in response to salt stress in *Nicotiana tabacum*

**DOI:** 10.1186/s12870-023-04659-0

**Published:** 2023-12-15

**Authors:** Zefeng Li, Huina Zhou, Guoyun Xu, Peipei Zhang, Niu Zhai, Qingxia Zheng, Pingping Liu, Lifeng Jin, Ge Bai, Hui Zhang

**Affiliations:** 1 China Tobacco Gene Research Center, Zhengzhou Tobacco Research Institute of CNTC, Zhengzhou, 45000 China; 2 Beijing Life Science Academy (BLSA), Beijing, China; 3 National Tobacco Genetic Engineering Research Center, Yunnan Academy of Tobacco Agricultural Sciences, Kunming, Yunnan China

**Keywords:** Tobacco, RNA-seq, lncRNA, Salt stress, Co-expression

## Abstract

**Background:**

Long noncoding RNAs (lncRNAs) have been shown to play important roles in the response of plants to various abiotic stresses, including drought, heat and salt stress. However, the identification and characterization of genome-wide salt-responsive lncRNAs in tobacco (*Nicotiana tabacum L.*) have been limited. Therefore, this study aimed to identify tobacco lncRNAs in roots and leaves in response to different durations of salt stress treatment.

**Results:**

A total of 5,831 lncRNAs were discovered, with 2,428 classified as differentially expressed lncRNAs (DElncRNAs) in response to salt stress. Among these, only 214 DElncRNAs were shared between the 2,147 DElncRNAs in roots and the 495 DElncRNAs in leaves. KEGG pathway enrichment analysis revealed that these DElncRNAs were primarily associated with pathways involved in starch and sucrose metabolism in roots and cysteine and methionine metabolism pathway in leaves. Furthermore, weighted gene co-expression network analysis (WGCNA) identified 15 co-expression modules, with four modules strongly linked to salt stress across different treatment durations (MEsalmon, MElightgreen, MEgreenyellow and MEdarkred). Additionally, an lncRNA-miRNA-mRNA network was constructed, incorporating several known salt-associated miRNAs such as miR156, miR169 and miR396.

**Conclusions:**

This study enhances our understanding of the role of lncRNAs in the response of tobacco to salt stress. It provides valuable information on co-expression networks of lncRNA and mRNAs, as well as networks of lncRNAs-miRNAs-mRNAs. These findings identify important candidate lncRNAs that warrant further investigation in the study of plant-environment interactions.

**Supplementary Information:**

The online version contains supplementary material available at 10.1186/s12870-023-04659-0.

## Introduction

Soil salinity is a severe problem worldwide. Approximately 6% of the global soil area is affected by salt stress [1]. For crop plants, salinity is one of the major abiotic stresses that often leads to yield reduction [2]. Salinity causes both osmotic and toxicity stress, affecting plant growth, development and metabolism. Halophytes have evolved a series of defense mechanisms in response to salt stress, such as salt exclusion, salt excretion and salt dilution [3, 4]. Most crop species (glycophytes) are salt sensitive and need to rebuild the homeostasis of cell ions, osmosis and redox balance to adapt to salt stress [5]. Salt tolerance in plants is a complex trait regulated by genetic, physiological and environmental factors. Uncovering and optimizing the salt tolerance of different plant species plays a crucial role in crop breeding to improve resistance on salinized agricultural land. With the advancement of whole-genome and transcriptome sequencing technologies, it has been discovered that over 75% of transcripts found in higher eukaryotic genomes do not have the ability to code for proteins and are known as noncoding sequences [6]. Among these sequences, there is a specific class called long noncoding RNAs (lncRNAs) that are longer than 200 nucleotides and without ability to code proteins [7].

In general, lncRNAs can be classified into three categories based on their genomic location and orientations relative to adjacent coding genes: intronic lncRNAs, intergenic lncRNAs (lincRNAs), and antisense lncRNAs [8]. In recent years, an increasing number of studies have shed light on the diverse roles of lncRNAs in plant growth and development [9]. LncRNAs have been found to play crucial roles in the regulation of seed germination and seedling growth. For example, when BoNR8 (a cabbage homolog of AtR8) was expressed in *Arabidopsis thaliana*, it strongly affected germination efficiency under ABA and salt stress conditions [10]. Moreover, lncRNAs have been implicated in flowering regulation and reproductive development. During vernalization, cold temperatures can induce the removal and addition of H3K27me3 modification on FLC, thereby inhibiting its expression and impacting flowering [11]. To date, three lncRNAs, namely COOLAIR [12], COLDAIR [13], and COLDWRAP [14], have been identified for their significant involvement in the silencing of the FLC gene [15]. Furthermore, lncRNAs have also been found to respond to various biotic and abiotic stresses [16]. For instance, the lncRNA ELENA1 is implicated in pattern-triggered immunity, while the lncRNA DRIR exhibits responsiveness to salt stress. Another lncRNA, SVK (SVALKA), has been identified as being responsive to cold stress in Arabidopsis*.*

The advent of high-throughput sequencing technology has facilitated the identification and screening of numerous potential lncRNAs in various plant species, including Arabidopsis [17], pear [18], pepper [19], tomato [20], wheat [21], peach [22] and many others. These studies have encompassed a wide range of biotic and abiotic stress conditions. Numerous studies have been conducted to investigate the response of lncRNAs to salt stress, resulting in the identification of several salt -responsive lncRNAs [20, 23–25]. For instance, in the case of duckweed (*Spirodela polyrhiza*), a total of 2,815 novel lncRNAs were discovered, with 6.6% of them showing differential expression under saline conditions [26]. Similarly, in birch (*Betula platyphylla* Suk.), 539 lncRNAs were recently identified, with one particular lncRNA called LncY1 being characterized for its ability to enhance salt tolerance by regulating BpMYB96 and BpCDF3 [27].

Tobacco (*Nicotiana tabacum L.*) is a commercially important species and serves as a crucial model plant for scientific research. In recent years, there has been great interest in understanding the molecular mechanisms underlying salt response in tobacco. Various factors, such as transcription factors (TFs) [28], ion transporters [29, 30], and miRNAs [31], have been demonstrated to be involved in response to salt stress. However, to date, no salt tolerance or sensitivity-related lncRNAs have been identified in tobacco. The mechanisms by which lncRNAs respond to salt stress and affect the uptake and transportation of Na^+^ or Cl^-^ in tobacco have not been thoroughly investigated. Therefore, it is vital to investigate the regulatory mechanisms of lncRNAs under salt stress.

In this study, we performed a comprehensive genome-wide identification and characterization of lncRNAs in roots and leaves of tobacco at different time points. The potential functions of the differentially expressed lncRNAs (DElncRNAs) and some key DElncRNAs were analyzed and obtained based on weighted gene co-expression network analysis (WGCNA) and lncRNA-miRNA-mRNA networks construction. These results would provide valuable information for understanding the function of lncRNAs in tobacco salt tolerance.

## Materials and methods

### Plant material and salt stress treatment

The tobacco cultivar ‘K326’ (*Nicotiana tabacum* L*.*) was chosen for this study, which was preserved in China Tobacco Gene Research Center. All plants were grown in the greenhouse in our lab. The salt stress experiment was conducted following the previously described procedure [30]. Briefly, seedlings were grown in plastic pots under a 16-hour photoperiod with temperatures of 28 ℃ during the day and 23 ℃ at night. To initiate salt treatment, plants at the six-leaf stage were transferred to a nutrient solution, as specified in the previous study [30], for a period of one week. Afterwards, a final concentration of 300 mM NaCl was added to the nutrient solution. Sampling was carried out at specific time points after the addition of NaCl: 3 hours, 6 hours, 12 hours, 24 hours, 3 days and 7days. Control samples were collected before the initiation of salt stress. The leaves and roots were separated from the plants, with the roots being rinsed thoroughly to remove any remaining nutrient solution and then dried gently. For each time point of salt treatment, triplicate samples were collected. All samples were immediately frozen in liquid nitrogen and stored at -80 °C until further analysis.

### Determination of MDA and proline content

The total malondialdehyde (MDA) content was determined using a modified thiobarbituric acid (TBA) method [32]. Approximately 0.5 g of leaf or root tissue was ground in 10 ml of pre-cooled PBS buffer (pH 7.8). The resulting homogenates were kept at 4 °C for 2 hours with intermittent shaking every 15 minutes. Afterward, the samples were centrifuged at 10,000 rpm for 20 minutes under low-temperature conditions. One milliliter of the supernatant was mixed with 1 ml of 10% TCA and 2 ml of 0.67% TBA, and the mixture was boiled for 25 minutes, rapidly cooled on ice, and then centrifuged again at 10,000 rpm for 5 minutes. The absorbance at 450 nm, 532 nm, and 600 nm was measured using a TECAN-Spark multimode microplate reader. Three biological replicates were performed.

Theproline contents were determined using a modified ninhydrin reaction method [32]. Leaf or root tissue (0.6 g) was homogenized in 6 ml pre-cooled 3% sulfosalicylic acid. The extracts were boiled for 20 minutes with intermittent shaking during the extraction process. The mixture was rapidly cooled on ice and then centrifuged at 4 ℃ and 10,000 rpm for 20 minutes. One milliliter of the supernatant was mixed with 1 ml of glacial acetic acid and 1ml of ninhydrin reagent, boiled for 30 minutes, rapidly cooled on ice, and then centrifuged again at 10,000 rpm for 5 minutes. The absorbance at 520 nm was measured using a TECAN-Spark multimode microplate reader. Three biological replicates were performed.

### RNA extraction, library establishment and sequencing

Total RNA extraction was carried out using the RNAprep pure Plant Kit (Tiangen, Beijing, China), following the manufacturer’s instructions. The quality of the extracted RNA was assessed by running it on a 1% agarose gel, and the purity was measured using a Nano Drop 2000 spetrophotometer. For library construction, 1 μl of the high-quality RNA was used. After the ribosomal RNA was eliminated, the remaining RNA were then fragmented and used for library preparation. Paired-end sequencing was performed using the Illumina NovaSeq 6000 System, generating the 150 bp length of paired-end reads. The raw sequence data have been deposited in the NCBI database under project ID PRJNA827645.

### Read preprocessing and mapping

To ensure the quality of the sequenced libraries, a series of quality control steps were performed using an in-house software ng_qc (Novogene). Raw data in fastq format (raw reads) was processed by an internal perl script. First, reads containing adaptors were removed from the dataset. Next, reads with N ratios exceeding 0.002 were discarded. Then, pair-end reads with more than 50% low-quality bases in either read were eliminated. Finally, the remaining high-quality, clean read sequences were aligned to the reference genome [33] of the tobacco cultivar ‘K326’ using HISAT2 (v2.1.0) [34]. The contents of Q20%, Q30%, GC%, ambiguous bases rate (Ns and percent per million), clean ratio ((clean data bases/raw data bases)*100%) were also calculated. This alignment step allowed for the mapping of the clean reads to the reference genome, enabling downstream analysis.

### Prediction of lncRNA

The transcripts from each sample were assembled individually using StringTie (v2.1.7) [35]. The StringTie-merge program was then utilized to generate a non-redundant set of transcripts. Cuffcompare [36] was employed to annotate the transcripts based on the obtained non-redundant set. For expression quantification, StringTie was used. To identify lncRNA, several criteria were applied for transcript screening: (1) the transcripts with annotation codes “i”, “x”, “u”, “o” or “e”; (2) transcripts length ≥200 bp; (3) number of exons≥2; and (4) expression level with FPKM ≥ 0.5. Additionally, the potential coding ability of the transcripts was assessed using four different software programs: CNCI (v2.0) [37], CPC2 (v0.1) [38], CPAT (v3.0.2) [39] and PfamScan (v1.3) [40]. To differentiate known and novel lncRNAs, lncRNA annotations from another tobacco genome (TN90) were collected from the NCBI database (https://www.ncbi.nlm.nih.gov/datasets/genome/GCF_000715135.1/). These TN90 lncRNAs were mapped to the reference genome using the GMAP program (version 2017-11-15) with the parameters --min-identity=0.95 and --min-trimmed-coverage=0.9. Cuffcompare was then used to compare the predicted lncRNAs against the TN90 lncRNAs. LncRNAs with class codes “=” or “c” were considered known, and others were classified as novel.

### Identification and analysis of differentially expressed lncRNAs and mRNAs

The control samples, prior to salt stress, were used as reference points for comparison. DESeq2 [41] was employed to identify the differentially expressed lncRNAs (DElncRNAs) and mRNAs (DEmRNAs) in response to salt stress. The DElncRNAs and DEmRNAs had an adjusted *p* value < 0.05 and | log2FC| ≥1. Clustering analysis of the expression profiles was conducted using Clust (v1.12.0) [42]. Gene ontology (GO) and Kyoto encyclopedia of genes and genomes (KEGG) pathway enrichment analyses were performed using clusterProfiler [43]. A significance cutoff of adjusted *p* value < 0.05 was used for identifying significantly enriched GO terms and pathways.

### Prediction of DElncRNA target genes

To perform functional annotation of the DElncRNAs, co-localization and co-expression analyses were conducted. In order to identify the target genes of DElncRNAs, Pearson’s correlation coefficient (r) was calculated to measure the expression level correlation between DElncRNAs and DEmRNAs. DEmRNAs with a correlation coefficient |r|>=0.9 and a *p-*value< 0.05 were selected as potential targets of DElncRNAs. To determine the *cis*-targets, a custom Python script was used to compare the genomic positions of DElncRNAs and DEmRNAs. DEmRNAs located within a distance of 100kb from the DElncRNAs were identified as *cis*-targets. For *trans*-target prediction, pRIblast software (v0.03) [44], a parallel version of RIblast [45], was utilized. The criteria for predicting *trans*-targets were an interaction energy of less than 14 kcal/mol and an interaction length ≥15 bp.

### Weighted Gene Co-Expression Network Analysis (WGCNA)

WGCNA [46] analysis was performed on both the DElncRNAs and DEmRNAs. An unsigned co-expression network was constructed based on their expression profiles. The soft-thresholding power was set to 8, which ensures a scale-free network. The minimum module size was set to 30, meaning that modules with fewer than 30 genes were not considered. A cutheight of 0.2 was used for merging close modules. For visualization of the network, the node and edge files for each module were exported and imported into Cytoscape software [47].

### LncRNA-miRNA-mRNA network construction

Published tobacco miRNAs from miRbase (Release 21, June 2014) were utilized to investigate the interactions in this study. The interactions between lncRNA-miRNA and mRNA-miRNA were predicted through the use of psRNATarget software [48], following the scoring schema V2 (2017 release). To identify potential lncRNA-mRNA pairs, the expression correlation between lncRNAs and mRNAs was examined. Pairs showing a correlation coefficient (|r|)>= 0.9 and a *p-*value< 0.05 was considered as potentially interacting pairs. Moreover, to construct lncRNA-miRNA-mRNA interaction networks, a custom Python script was developed and employed. The networks were then visualized using Cytoscape software.

### qRT-PCR validation

In this study, total RNA was extracted using the RNAprep Pure Plant Kit (Tiangen, Beijing, China), following the instructions provided [29]. Subsequently, the RNA was reverse transcribed into cDNA using the Transcriptor First Strand cDNA Synthesis Kit (Roche). For qRT-PCR of lncRNAs, SYBR Green premix (2×) (Roche) was used, and the reactions were performed on a LightCycler ® 96 Real-Time PCR System (Roche). To ensure data accuracy, the *Nt26S* gene was employed as the reference gene. The PCR cycling program consisted of an initial incubation step at 95 °C for 10 minutes, followed by 40 cycles of denaturation at 95 °C for 10 seconds, annealing at 58 °C for 20 seconds, and extension at 72 °C for 20 seconds. All the primer sequences used for qRT-PCR are listed in Supplementary Table S1.

## Results

### Physiological characterization of tobacco in response to salt stress

**Fig. 1:**
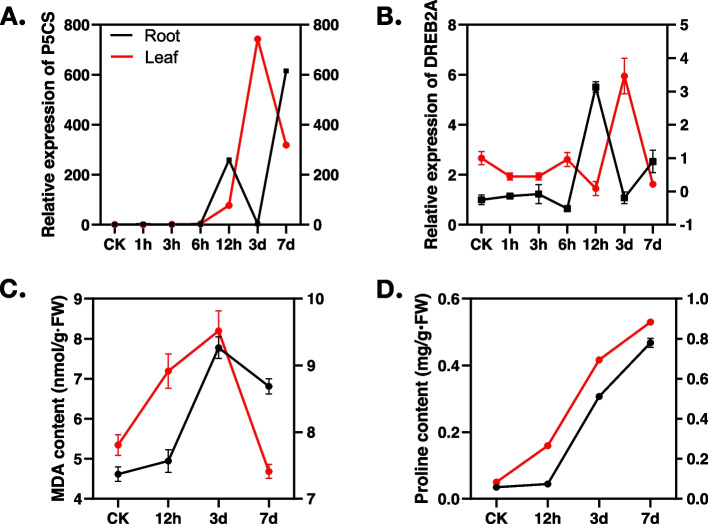
Analysis of gene expression, proline and MDA content in tobacco roots and leaves under different salt treatment durations. Panels **A** and **B** represent expression changes in *P5CS* and *DREB2A*, respectively. Panels **C** and **D** represent proline and MDA contents, respectively

To comprehensively investigate the impact of salt stress on tobacco, we conducted a study that included multiple time points to capture both early-stage and long-term responses. Specifically, we selected 1 hour, 3 hours, 6 hours, and 12 hours as representative time points for the early stage responses to salt stress, while 24 hours, 3 days, and 7 days represented the long-term responses. In order to determine the key time points for salt stress responses, we examined the expression of two known stress-responsive genes, *P5CS* and *DREB2A* [49]. As depicted in Fig. 1A and B, both genes were significantly induced at 12 hours in the roots and at 3 days in the leaves. Additionally, the expression of *P5CS* showed another increase at 7 days in the roots. Hence, 12 hours, 3 days and 7 days were selected for the subsequent sequencing experiment. Furthermore, we measured the contents of MDA and proline (Fig. 1C and D). The MDA content increased during the first 3 days of salt treatment but decreased at 7 days in both roots and leaves. On the other hand, the proline contents continued to rise throughout the duration of the salt stress in both roots and leaves.

### Whole transcriptome sequencing analysis of different samples

To investigate the response of lncRNAs in cultivated tobacco under salt stress, we collected roots and leaves from plants that had been subjected to salt stress for different durations (12 hours, 3 days, and 7 days). Whole transcriptome sequencing analysis was conducted with three replicates for each sample. In total, the Illumina platform generated approximately 2,217 million raw reads. After removing low-quality sequences, we obtained 2,185 million clean reads (Supplementary Table S2). The clean rate of clean reads reached 98.52%, with an average of around 91 million reads per sample. Through mapping these clean reads to the reference genome, we achieved alignment rates ranging from 83% to 98% for each sample, with unique mapped rates between 77% and 88% (Table 1).

**Table 1:** Statistical qualification of high throughput sequencing data

**Sample**	**Raw reads**	**Clean reads**	**Mapped**	**Uniquely mapped**
RCK-1	78493782	76715372	69240868(90.26%)	62395517(81.33%)
RCK-2	90652286	89528660	80175991(89.55%)	74115789(82.78%)
RCK-3	83489260	82312440	73990643(89.89%)	68268149(82.94%)
R12h-1	85202208	84101512	78435918(93.26%)	72423156(86.11%)
R12h-2	88640876	87377838	79468781(90.95%)	73413593(84.02%)
R12h-3	86437516	85094334	76240385(89.6%)	70287421(82.6%)
R3d-1	95305884	93888676	82728419(88.11%)	76105824(81.06%)
R3d-2	100594106	99110234	93446449(94.29%)	86215575(86.99%)
R3d-3	85991280	84806168	71124300(83.87%)	65374090(77.09%)
R7d-1	86545194	85415952	77896434(91.2%)	71575984(83.8%)
R7d-2	88638568	87624378	83018430(94.74%)	76613532(87.43%)
R7d-3	84833002	83686042	79242374(94.69%)	72988336(87.22%)
LCK-1	91605898	90042102	87505706(97.18%)	75753072(84.13%)
LCK-2	95443466	94230186	90449338(95.99%)	81250537(86.23%)
LCK-3	100102960	98156830	95110331(96.90%)	83578793(85.15%)
L12h-1	106122454	104127380	100634471(96.65%)	88417828(84.91%)
L12h-2	101236356	99884132	96678666(96.79%)	84497881(84.60%)
L12h-3	96617204	94803582	91850228(96.88%)	81047828(85.49%)
L3d-1	99983868	98805920	95909622(97.07%)	83805871(84.82%)
L3d-2	93316850	91999278	89195563(96.95%)	77161824(83.87%)
L3d-3	103412860	101716588	98694012(97.03%)	85989070(84.54%)
L7d-1	100040082	98593216	95527067(96.89%)	82910848(84.09%)
L7d-2	86846072	85786174	82748442(96.46%)	74186582(86.48%)
L7d-3	87868758	86879714	84326691(97.06%)	73351819(84.43%)

### Identification of lncRNAs and mRNAs in tobacco

**Fig. 2:**
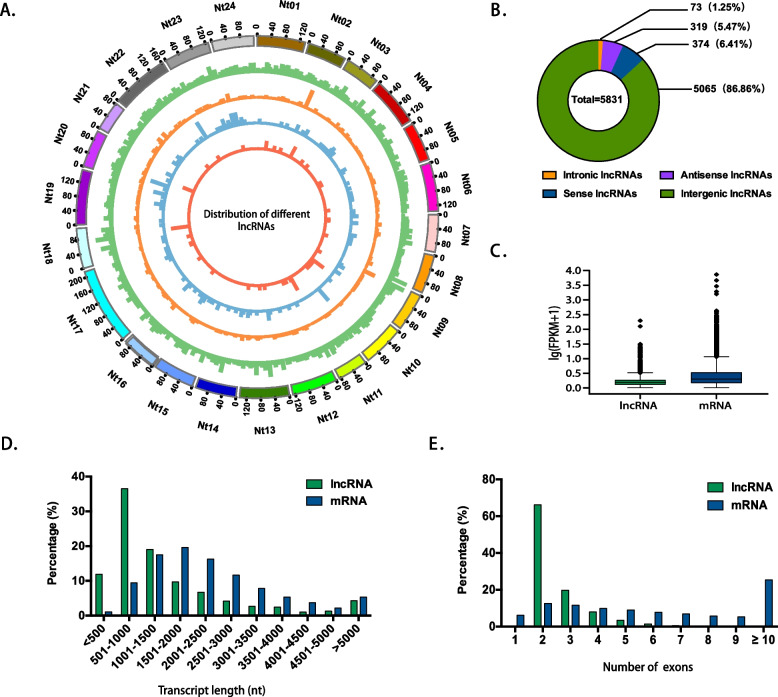
Comparison of structural features between lncRNAs and mRNAs. **A** Chromosomal distribution of different types of lncRNAs. **B** Classification of identified lncRNAs. **C** Expression level comparison between lncRNAs and mRNAs. **D** Length distribution of lncRNAs and mRNAs. **E** Exons distribution inlncRNAs and mRNAs. In panels C to E, green represents lncRNAs, and blue represents mRNAs

In our study, a total of 5,831 lncRNAs (Supplementary Table S3) and 52,704 mRNAs (Supplementary Table S4) were identified. Among the lncRNAs, 5,065 (86.86%) were categorized as intergenic lncRNAs, 374 (6.41%) were sense lncRNAs, 319 (5.47%) were antisense lncRNAs and 73 (1.25%) were intronic lncRNAs based on their locations within the *N. tabacum* genome (Fig. 2B, Supplementary Table S5). By comparing our findings with previously known lncRNAs, we identified a total of 1,219 known lncRNAs and 4,612 novel lncRNAs (Supplementary Table S6). The distribution analysis revealed that lncRNAs were evenly distributed across all 24 chromosomes of tobacco (Fig. 2A). In comparison to mRNAs, lncRNAs generally exhibited lower expression levels (Fig. 2C). The average transcript length of the lncRNAs was shorter than that of the mRNAs (Fig. 2D). Specifically, 55.6% of lncRNAs shared a length of 500 bp to 1,500 bp, while 53.4% of mRNAs had a length of 1,000 bp to 2,500 bp. Furthermore, most lncRNAs shared 2 or 3 exons (Fig. 2E). Specifically, 3,855 lncRNAs, accounting for 66.1% of total identified lncRNAs, had 2 exons, while 1,153 lncRNAs (19.8%) had 3 exons. In contrast, mRNAs typically had more exons, with 25.4%, 44.3%, and 24.1% of mRNAs having over 10, 4-9, and 2-3 exons, respectively. These differences in exon numbers may indicate or correlate with distinct functions between lncRNAs and mRNAs.

### Identification of differentially expressed lncRNAs (DElncRNAs) responsive to salt stress

**Fig. 3:**
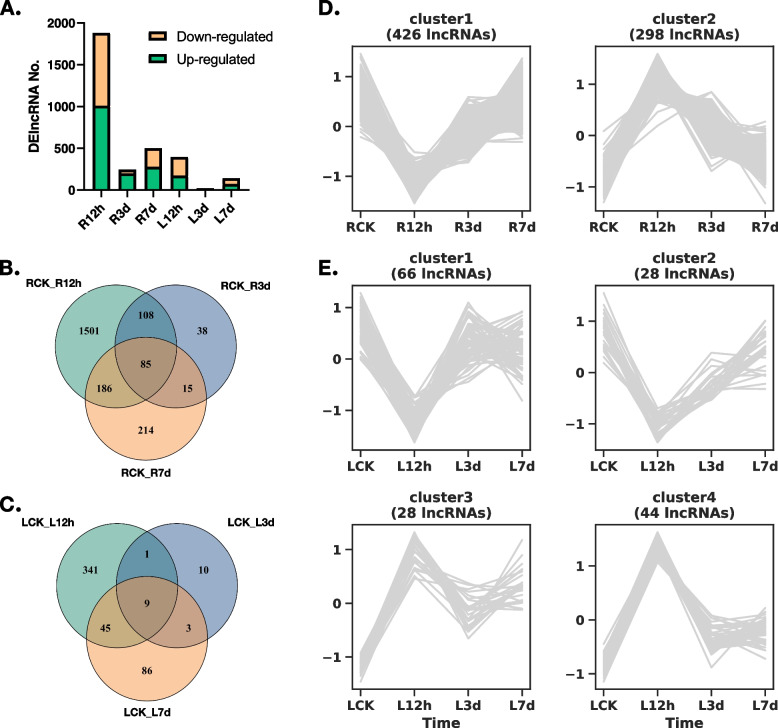
Transcriptomic profiling of tobacco lncRNAs in response to salt stress. **A** Number of down-regulated and up-regulated lncRNAs at different time points of salt treatment, compared to samples before salt treatment. Venn diagrams of the DElncRNAs in roots (**B**) and leaves (**C**) at three different time points. Clustering analysis of DElncRNAs in roots (**D**) and leaves (**E**)

Through pairwise comparison with K326 grown under normal conditions, a total of 2,147 differentially expressed lncRNAs (DElncRNAs) in the roots and 495 DElncRNAs in the leaves in response to salt stress were identified. Among these, 214 DElncRNAs were found to be shared between the roots and leaves. As indicated in Supplementary Table S7 and Fig. 3A, the majority of the DElncRNAs (1,880 out of 2,147) in the roots and 396 out of 495 DElncRNAs in the leaves exhibited significant expression changes after 12 hours of salt stress compared to longer durations of 3 days or 7 days of salt stress. Regarding the detected three time points of salt stress, only a small subset of DElncRNAs (85, accounting for 4.0% in roots, and 9, accounting for 1.8% in leaves) consistently showed significant regulation throughout all time points (Fig. 3B and C).

Furthermore, we conducted clustering analysis to categorize the expression profiles of DElncRNAs in the roots and leaves. In the roots, the DElncRNAs were classified into two distinct groups. While in the leaves, they were classified into four groups (Fig. 3D and E). Interestingly, we observed a remarkably similar pattern of response to salt stress between DElncRNAs in the roots and leaves, suggesting a potential correlation between lncRNA expression and the response to salt stress.

### Target gene prediction and functional annotation of DElncRNAs

To gain insights into the function of DElncRNAs, we analyzed their potential *cis*- and *trans*- target mRNAs. In the roots, we found that 78 DElncRNAs regulated 86 *cis*-target mRNAs and 1,814 DElncRNAs regulated 12,656 *trans*-target mRNAs. In the leaves, 6 DElncRNAs regulated 6 *cis*-target mRNAs and 353 DElncRNAs regulated 3,222 *trans*-target mRNAs.

**Fig. 4:**
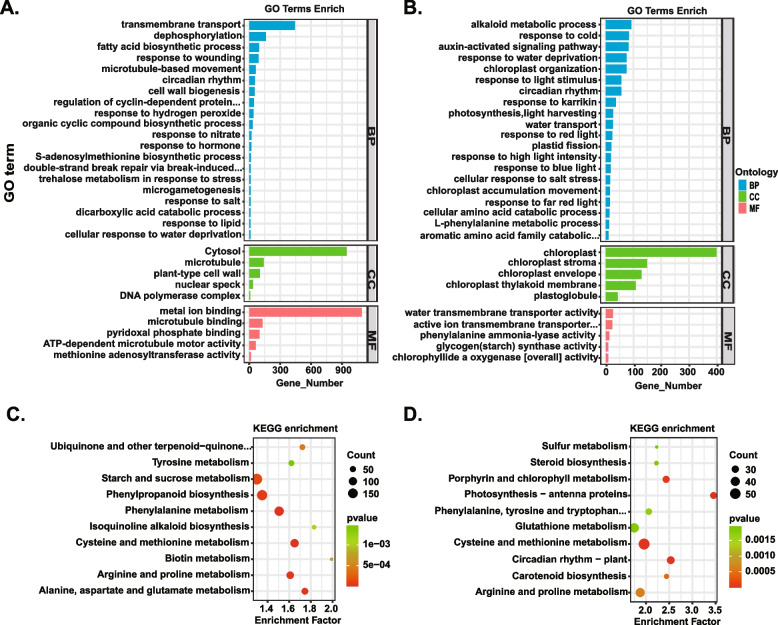
Functional annotation of differentially expressed lncRNAs by GO classification (**A** & **B**) and KEGG enrichment (**C** & **D**) in roots (**A** & **C**) and leaves (**B** & **D**). The top 20 significant GO terms in the biological process category and the top 5 significant GO terms in the cellular component and molecular function categories were selected based on the cutoff of *p* adjust < 0.05. The top 10 KEGG enrichment pathways were selected based on the cutoff of *p* adjust < 0.05. The KEGG pathway map was sourced from KEGG Mapper (https://www.kegg.jp/kegg/mapper/), and we have obtained written permission to use and adapt it [50–52]

Additionally, we performed GO and KEGG enrichment analyses for all the DElncRNAs. In the root DElncRNAs, GO analysis revealed that translation in biological processes, cytosol in cellular components, and structural constituent of ribosome in molecular functions were the most enriched GO terms (Fig. 4A). These results were consistent with the crucial role of translation and ribosome function in roots during salt stress. In the leaf DElncRNAs, the most enriched GO terms across all three categories were chloroplast organization in biological processes, chloroplast in cellular components, and mRNA binding in molecular functions (Fig. 4B). This suggests that chloroplasts may play a significant role in the response to salt stress in tobacco leaves.

Further KEGG analysis showed that the most significantly enriched pathways in the roots were mainly related to C/N metabolism, such as starch and sucrose metabolism, cysteine and methionine metabolism, arginine and proline metabolism (Fig. 4C). Similarly, the most significantly enriched KEGG pathways in the leaves were also related to C/N metabolism in leaves, such as porphyrin and chlorophyll metabolism, circadian rhythm-plant, cysteine and methionine metabolism, fructose and mannose metabolism, and arginine and proline metabolism (Fig. 4D and Supplementary Table S8).

### Screening of lncRNAs and genes related to salt stress by WGCNA

**Fig. 5:**
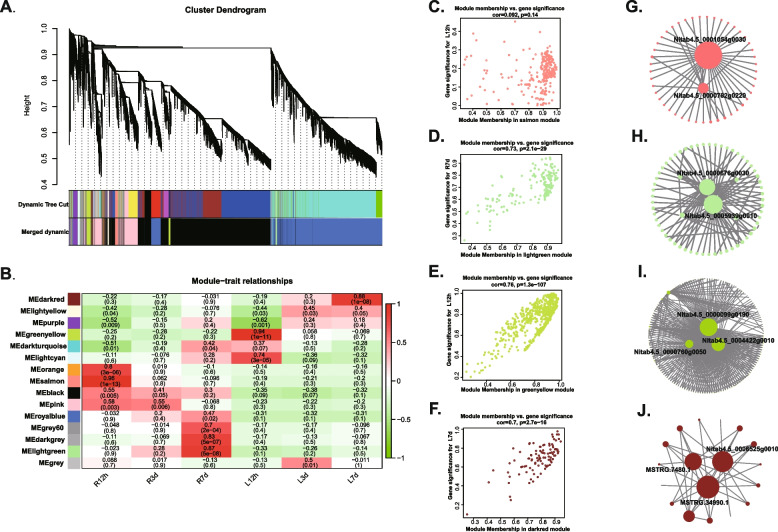
WGCNA of genes and lncRNAs in tobacco roots and leaves under salt treatment. **A** Transcripts hierarchical clustering tree of different modules. Each major tree branch represents one module, each leaf in the tree represents one transcript, and different modules are labeled with different colors. **B** Module-trait relationship. Each row represents a module eigengene, and each column presents a trait. The coefficient and *p* value of the correlation between each module and trait are shown. **C**-**F** Scatter plots of transcript significance (TS) versus module membership (MM) of the transcripts in the four exemplified salt-associated modules (MEsalmon, MElightgreen, MEgreenyellow and MEdarkred). **G**-**J** Gene networks and hub nodes involved in the four salt-associated modules

WGCNA was conducted using different time points of salt treatment as phenotypic information to construct co-expression modules of lncRNAs and genes. A total of 15 co-expression modules were generated (Fig. 5A and Supplementary Table S9). To identify salt stress-related modules, the correlation between gene modules and phenotypes was calculated (Fig. 5B). Four modules, MEsalmon, MElightgreen, MEgreenyellow and MEdarkred, were found to be significantly associated with 12 hours and 7 days salt stress in roots (R12h and R7d) and leaves (L12h and L7d), respectively. The eigengene network further confirms the relationships among the four modules and stress conditions in roots and leaves. Scatter plots (Fig. 5C-F) revealed a strong positive correlation between transcript significance (TS) and module membership (MM).

Additionally, we selected genes with higher weight (above 0.35 in R12h and L12h, and above 0.25 in R7d and L7d) in each module for network construction and analysis (Fig. 5G-J). In the MEsalmon module, two hub genes (*Ntab4.5_0001054g0030* and *Ntab4.5_0000782g0220*) were identified. *Ntab4.5_0001054g0030* encodes an E3 ubiquitin-protein ligase (UBL), which is known to play important roles in responding to abiotic stresses like drought [53] and salt stress [54]. In the MElightgreen module, two hub genes (*Nitab4.5_0000676g0030* and *Nitab4.5_0005939g0010*), both encoding unknown proteins, were selected. In the MEgreenyellow module, three hub genes (*Nitab4.5_0000099g0190*, *Nitab4.5_0000760g0050* and *Nitab4.5_0004422g0010*) were chosen, encoding axanthoxin dehydrogenase, a cytochrome P450 and an auxin-binding protein, respectively. In the MEdarkred module, two lncRNAs (MSTRG.34990.1 and MSTRG.7480.1) and *Nitab4.5_0006525g0010* were identified as hub nodes. *Nitab4.5_0006525g0010* encoded an F-box protein, which has been associated with the response to salt stress [55].These findings indicate that hub genes or hub lncRNAs may play important roles under salt stress conditions, either in roots or in leaves.

### LncRNA-miRNA-mRNA networks under salt stress

**Fig. 6:**
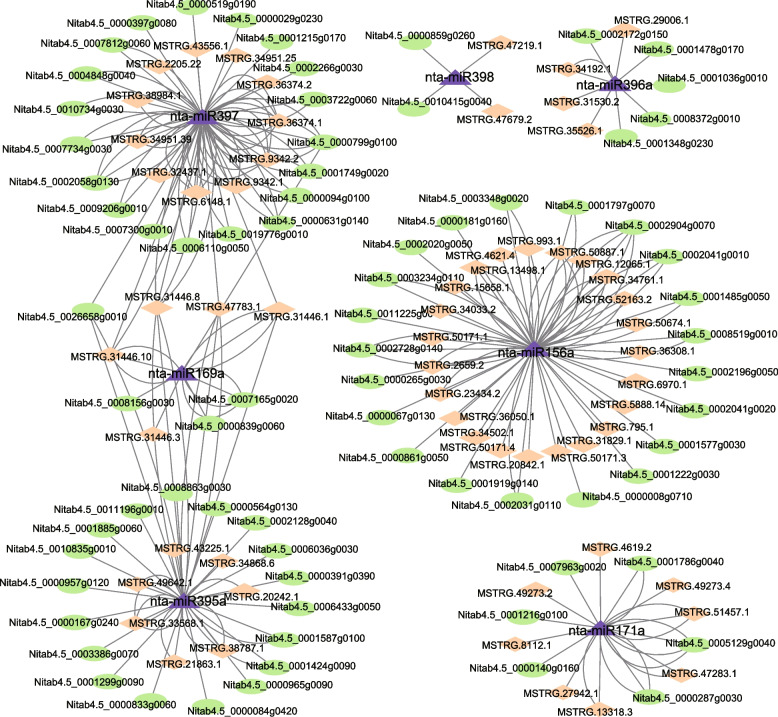
LncRNA-miRNA-mRNA co-expression network. Diamond indicates lncRNA, triangle indicates miRNA, and ellipse indicates mRNA

LncRNAs not only have the ability to regulate mRNAs in a *trans* or *cis* manner, but they can also function as competitive targets for miRNAs, thereby influencing the regulatory efficiency of these miRNAs. One approach to assess the relationship between lncRNAs and miRNAs is by utilizing lncRNAs as endogenous target mimics (eTMs) for miRNAs. In this study, we identified 774 DElncRNAs in tobacco roots that are involved in the regulation of 2,488 mRNAs through interactions with 162 miRNAs. Similarly in tobacco leaves, we found 139 DElncRNAs that regulate 556 mRNAs through interactions with 121 miRNAs (Supplementary Table S10). Notably, several miRNAs, such as miR156 [56], miR169 [57], miR171 [58], miR386 [59], miR397 [60] and miR398 [61], have been previously implicated in the response to salt stress, and we also observed their presence in our constructed lncRNA-miRNA-mRNA networks in roots, exhibiting a strong correlation of 0.95 (Fig. 6). For instance, nta-miR156a was associated with 44 targets, including 21 mRNAs and 23 lncRNAs, whereas nta-miR171a had 14 targets, consisting of 6 mRNAs and 8 lncRNAs. It is worth noting that multiple miRNAs can simultaneously regulate the same target. An example of this is seen with the coordinated regulation of the expression of *MSTRG.31446.1* by nta-miR169a, nta-miR395a and nta-miR397.

### Validation of target genes of salt-responsive lncRNAs

**Fig. 7:**
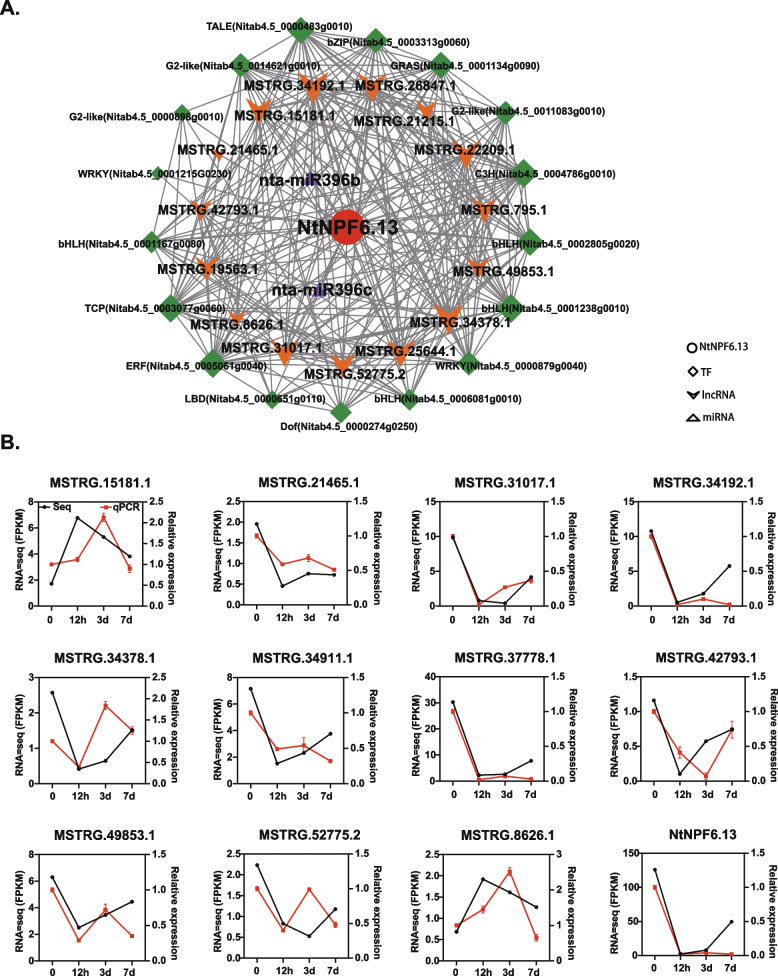
Calculated interaction network of *NtNPF6.13* (**A**) and gene expression validation of *NtNPF6.13* and its associated lncRNAs. For panel **B**, the black line with a solid circle represents the RNA-seq results, and the red line with a solid box represents the qRT-PCR results. The error bars represent the standard error of 3 replicates

In our previous study, we have demonstrated the involvement of *NtNPF6.13* in the response to salt stress in tobacco. In this study, we constructed a co-expression network of TFs, lncRNAs and miRNAs associated with *NtNPF6.13* (Fig. 7A). Within this network, we identified a total of 17 TFs (such as bHLH, WRKY and ERF), 15 lncRNAs and 2 miRNAs (nta-miR396b and nta-miR396c). To validate the accuracy of the sequencing data, we performed qRT-PCR to examine the expression patterns of *NtNPF6.13* and 11 lncRNAs (the other 4 lncRNAs were not detected in qRT-PCR). As shown in Fig. 7B, there is a strong agreement between the RNA-seq results and qRT-PCR data for *NtNPF6.13* and most of the tested lncRNAs, particularly at the 12-hour time point. This supports the reliability of the sequencing data and the subsequent analysis results. Interestingly, two lncRNAs, namely MSTRG.34192.1 and MSTRG.37778.1, exhibited nearly identical expression patterns to *NtNPF6.13*. Further experiments are required to confirm their relationship with *NtNPF6.13* and to elucidate their roles in the response to salt stress.

## Discussion

Despite extensive research on lncRNAs in various plant species, the exploration of lncRNAs in tobacco is still in its early stages. Previous studies have investigated tobacco lncRNAs under root-knot nematode stress [62], herbivore stress [63], nicotine pathway [64] and axillary bud development [65]. However, there is a notable dearth of studies focused on the identification of tobacco lncRNAs responsive to salt stress. In this study, we conducted an investigation to analyze the expression of lncRNAs in tobacco roots and leaves under salt stress at various treatment time points by employing whole transcriptome sequencing. And 2,147 and 495 DElncRNAs were identified in tobacco roots and leaves, respectively. It has been reported that roots of both the wild tomato *Solanum pennellii* and cultivated tomato M82, belonging to the same Solanaceae family as *N*. *tabacum,* exhibited 154 and 137 DElncRNAs respectively [20]. The significant difference in the number of DElncRNA between tobacco and previous reports may be attributed to variations in plant species, ploidy level, salt stress duration, or the specific filter criteria applied for lncRNA identification.

Despite the observed diversity among different plant species and stress conditions, lncRNAs generally display certain fundamental characteristics, including a relatively short sequence length, low expression level, and a predominance of 1 or 2 exons [66]. Consistent with the previous findings in peach and other plants [22, 27], our study found that the majority of lncRNAs in tobacco were less than 1,000 nt in length and consisted of only 2-3 exons. In comparison to previous studies that identified salt-stressed lncRNAs in other plants [20, 23, 24, 26, 27], our study considered different time points post salt stress, ranging from the early-stage (12 hours) to the long-term stage (7 days) in both roots and leaves. Notably, the number of DElncRNAs in the roots was more than four times higher than that in the leaves. Particularly, the highest number of DElncRNAs was observed at the early-stage of salt stress treatment. Furthermore, our findings revealed that some DElncRNAs were expressed at a single time point, while others were expressed at multiple time points (Supplementary Figure S1). This dynamic expression pattern suggests that DElncRNAs may play a role in regulating salt stress in a more dynamic manner and time-dependent manner. These results strongly support the notion that the expression of salt–responsive lncRNAs in tobacco is tightly regulated in a tissue-specific and temporal-dependent manner, in accordance with previous reports in duckweed [26].

WGCNA is a commonly employed method in systems biology that allows for the identification of gene modules displaying co-expression patterns. It also enables the investigation of the relationship between these modules and phenotypic data [46]. It was observed that tobacco plants exhibited wilting at 12 hours under salt stress, indicating a significant influence of salt stress at this time point (data not shown). After the plants adapted to the salt stress for 3 days, they were able to grow normally but were slightly smaller than the controls. However, after 7 days of salt stress, the plant growth was significantly inhibited, suggesting that long-term responses to salt stress involve specific genes. In line with the growth conditions, our study identified four distinct salt-associated modules at the early-stage (12 hours) and long-term stage (7 days) of salt stress both in roots and leaves (Fig. 5C-F). Previous research in *Populus trichocarpa* reported the identification of six salt-responsive modules using WGCNA in different tissues (leaf, stem and root) under short-term (24 hours) and long-term (7 days) salt stress [67]. Interestingly, the correlations among the modules were significantly lower in the short-term salt stress compared to the long-term salt stress, indicating a more pronounced response to long-term salt treatment in Populus. In our study, the correlation values of modules in both the 12 hours and 7 days salt treatment were higher. Additionally, the correlation values of modules in long-term salt treatment were slightly lower than those in the short- term treatment. In our study, several hub genes and lncRNAs within the salt-responsive modules were identified. However, it is interesting to note that no TFs were identified as hub genes in contrast to previous studies.

LncRNAs are known to interact with miRNAs in various ways, such as serving as miRNA precursors, target mimics or direct targets in response to abiotic stress [6]. Among the plant miRNA families, miR169 is the largest and most conserved miRNA family [68]. It typically targets members of the NF-YA transcription factor gene family, playing a crucial role in plant abiotic stress resistance. In this study, the NF-YA transcription factor Nitab4.5_0007165g0020 was found to be associated with nta-miR169. Interestingly, two lncRNAs (MSTRG.31446.1 and MSTRG.31446.10) were also identified interacting with nta-miR169. However, the exact mechanism of their interaction needs to be further investigated.

*NtNPF6.13,* a gene involved in chloride uptake, was found to be significantly down-regulated after salt stress [30]. To further investigate the function of *NtNPF6.13*, a co-expression network was constructed (Fig. 7A). Interestingly, two members of the nta-miR396 family were identified as targeting *NtNPF6.13* within this network. The role of miR396 in plant growth and development has been extensively studied. Over-expressing of miR396 in tobacco has been shown to lead to cotyledon fusion and the absence of a shoot apical meristem [69]. Furthermore, miR396 has also been reported to play a role in the response to salinity stress, particularly in the regulation of the Na^+^ transporter SOS1 in creeping bentgrass [59]. However, it is still not determined whether miR396 is involved in regulating the Cl^-^ transportation by NtNPF6.13 in tobacco. Further investigation is needed to provide evidence for this hypothesis. In addition, 17 TFs were identified in this co-expression network, including bHLH, WRKY and ERF. Notably, the transcription factor MtNLP1 has been shown to be essential for the regulation of MtNPF6.5, which mediates chloride uptake and preference in *Medicago* roots [70]. Consequently, it would be intriguing to explore and identify potential transcription factors that may play a role in regulating NtNPF6.13 and its involvement in chloride uptake in tobacco. Furthermore, during the validation of *NtNPF6.13* co-expressed lncRNAs, it was observed that MSTRG.34192.1 or MSTRG.37778.1 exhibited a similar expression pattern as *NtNPF6.13*. Further investigation into the specific roles of MSTRG.34192.1 or MSTRG.37778.1 in the tobacco salt stress would be valuable.

## Conclusions

In summary, a comprehensive analysis of lncRNAs involved in the salt stress response in tobacco was conducted. A total of 5,831 lncRNAs were identified, with 2,428 of them being differentially expressed in response to salt stress. KEGG pathway enrichment analysis highlighted the involvement of starch and sucrose metabolism pathways in the salt stress response of tobacco roots. The WGCNA analysis helped in identifying hub genes and lncRNAs associated with salt stress. Furthermore, the lncRNA-miRNA-mRNA network provided insights into the regulatory mechanism underlying salt stress in tobacco and identified potential candidate genes for enhancing salt stress tolerance in tobacco. This study contributes valuable information about the roles of lncRNAs in the salt stress response of tobacco. However, further functional analysis is necessary to validate the findings and elucidate the precise mechanisms by which these lncRNAs function in salt stress tolerance.

### Supplementary Information

**Additional file 1: Table S1.** Sequences of qPCR primers.

**Additional file 2: Table S2.** Raw data and clean data statistics.

**Additional file 3: Table S3.** FPKM of lncRNAs.

**Additional file 4: Table S4.** FPKM of mRNAs.

**Additional file 5: Table S5.** A list of lncRNA types.

**Additional file 6: Table S6.** A list of known lncRNAs and novel lncRNAs.

**Additional file 7: Table S7.** Lists of DElncRNAs in roots and leaves.

**Additional file 8: Table S8.** Lists of GO and KEGG enrichment in roots and leaves.

**Additional file 9: Table S9.** Gene significance and module membership.

**Additional file 10: Table S10.** Lists of lncRNA-miRNA-mRNA interaction triples in roots and leaves.

**Additional file 11: Figure S1.** KEGG pathway analysis of DElncRNAs at different time points in roots (A) and leaves (B) under salt stress.

## Data Availability

The datasets presented in this study can be found in online repositories. The address of the repository/repositories and accession number(s) can be found below: https://www.ncbi.nlm.nih.gov/, PRJNA827645.

## Abbreviations

LncRNA: Long noncoding RNA
KEGG: Kyoto Encyclopedia of Genes and Genomes
GO: Gene Ontology
DElncRNAs: Differentially expressed lncRNAs
DEmRNAs: Differentially expressed mRNAs
WGCNA: Weighted Gene Co-expression Network Analysis
