# Supplementary material for: Genome-wide analysis of long noncoding RNAs in response to salt stress in Nicotiana tabacum
Source: BMC Plant Biol. 2023 Dec 15;23:646. doi: 10.1186/s12870-023-04659-0 (PMC10722832; doi:10.1186/s12870-023-04659-0)
Supplement: Supplementary file 11 — Additional file 11: Figure S1. KEGG pathway analysis of DElncRNAs at different time points in roots (A) and leaves (B) under salt stress. [file 12870_2023_4659_MOESM11_ESM.pdf]

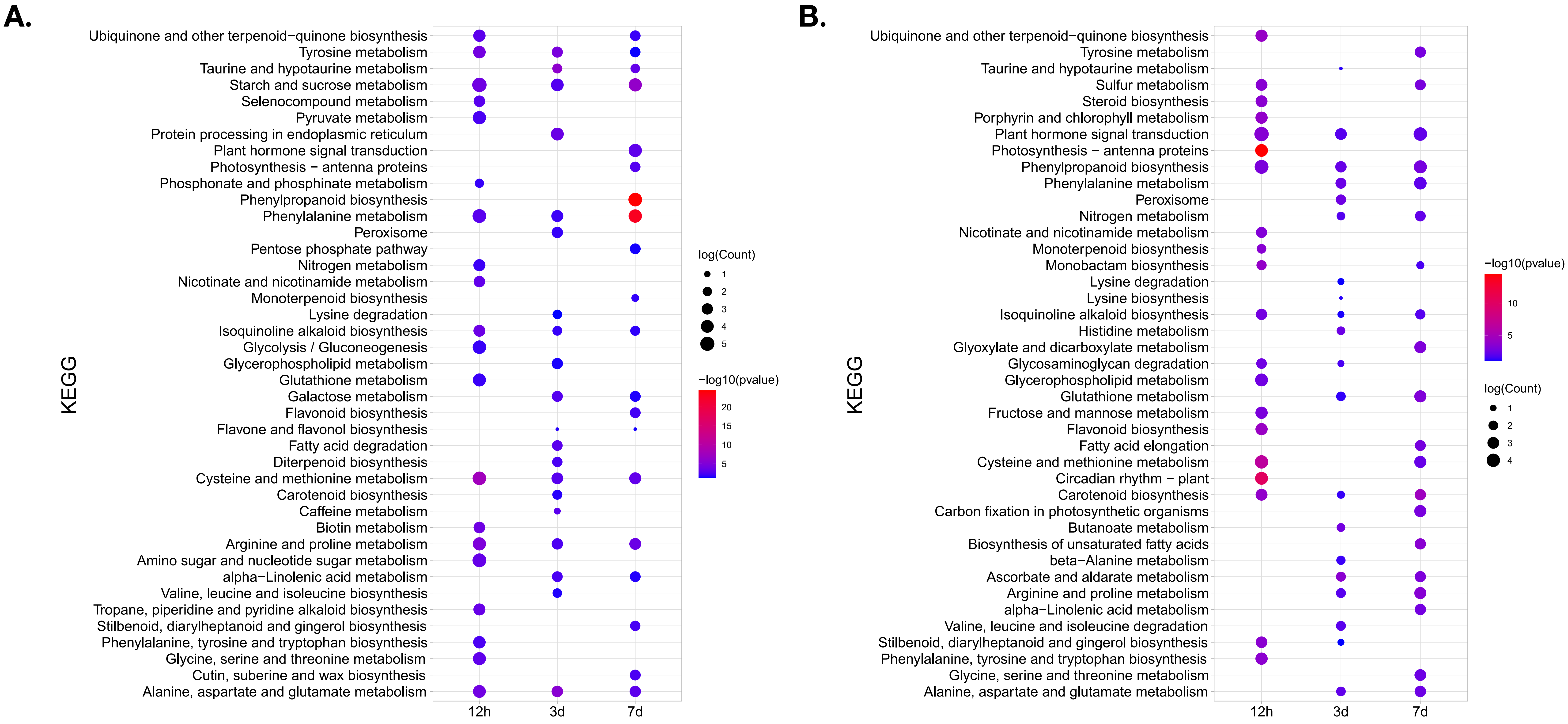

Figure S1. KEGG pathway analysis of DElncRNAs at different time points in roots (A) and leaves (B) under salt stress.
